# Over-Expression of the Heat-Responsive Wheat Gene *TaHSP23.9* in Transgenic *Arabidopsis* Conferred Tolerance to Heat and Salt Stress

**DOI:** 10.3389/fpls.2020.00243

**Published:** 2020-03-06

**Authors:** Jun Wang, Xin Gao, Jun Dong, Xinyu Tian, Junzhe Wang, Jairo A. Palta, Shengbao Xu, Yan Fang, Zhonghua Wang

**Affiliations:** ^1^State Key Laboratory of Crop Stress Biology for Arid Areas, College of Agronomy, Northwest A&F University, Yangling, China; ^2^CSIRO Agriculture and Food, Wembley, WA, Australia; ^3^The UWA Institute of Agriculture and School of Agriculture and Environment, The University of Western Australia, Perth, WA, Australia; ^4^State Key Laboratory of Soil Erosion and Dryland Farming on the Loess Plateau, Northwest A&F University, Yangling, China

**Keywords:** *TaHSP23.9*, small heat shock protein, heat stress, salt stress, wheat (*Triticum aestivum* L.), *Arabidopsis* (*Arabidopsis thaliana*)

## Abstract

The small heat shock proteins (sHSP) are stress-induced proteins with molecular weights ranging from 12 to 42 kDa that act as molecular chaperones to prevent the irreversible aggregation of denaturing proteins. In this study, we cloned the heat responsive gene *TaHSP23.9* from wheat (*Triticum aestivum*) based on TMT-labeled quantitative proteomic analysis in our previous work and examined its function in the response of transgenic *Arabidopsis* to heat and salt stress. Amino acid alignment and phylogenetic tree analysis showed that TaHSP23.9 contained a typically conserved structure of the alpha-crystallin domain and is closely related to OsHSP23.2 in rice. Transient expression assays demonstrated that TaHSP23.9 is located on the endoplasmic reticulum. Quantitative real-time PCR demonstrated that *TaHSP23.9* was expressed much more in filling grains under normal conditions and was significantly upregulated by heat and salt stress. Transgenic *Arabidopsis* plants that constitutively over-expressed *TaHSP23.9* had no visible differences or adverse phenotypes compared with the wild type under normal conditions but exhibited enhanced tolerance to heat and salt stress under stress conditions. In addition, we found that the expression level of *TaHSP23.9* was significantly higher in the heat-tolerant wheat varieties than in the heat-sensitive varieties. Our results suggest that TaHSP23.9 may function as a protein chaperone to positively regulate plant responses to heat and salt stress and could be developed as a molecular marker for screening heat-tolerant wheat varieties.

## Introduction

Wheat (*Triticum aestivum*) is one of the most important staple crops and is extensively cultivated all over the world (Lobell et al., 2011). As wheat is a chimonophilous crop, global warming presents a challenge for wheat production. Most wheat is adversely affected by temperatures above 35°C, especially during grain filling, which shorten the duration of grain filling and reduce grain yield and quality (Lesk et al., 2016; Zhao et al., 2017). Selecting thermotolerant wheat varieties by traditional breeding is difficult because heat tolerance in wheat is a complex quantitative trait that is controlled by multiple genes and is vulnerable to environmental factors (Paliwal et al., 2012; Talukder et al., 2014). Additionally, there are few direct and stable evaluation indicators and no evaluation index systems. Therefore, much attention should be given to using molecular biology to understand the networks within the plant heat stress response (HSR) and to breeding new thermotolerant wheat varieties through genetic engineering to cope with the yield loss and poor quality caused by high temperatures and heat waves (Fragkostefanakis et al., 2015; Ohama et al., 2017).

Under heat stress (HS), a series of physiological and biochemical reactions occur in organisms, and normal protein synthesis is inhibited. At the same time, many new proteins are rapidly synthesized to maintain the physiological balance inside the cells. The most important of these new proteins are the heat shock proteins (HSPs). They are a class of conserved proteins that act as molecular chaperones and not only combine with the polypeptides being synthesized and assist them in folding correctly but also prevent the irreversible aggregation of denaturing proteins and maintain or recover their biological functions against environmental stresses (Bukau et al., 2006; Papsdorf and Richter, 2014). HSPs can be divided into five major groups based on molecular weight: HSP100, HSP90, HSP70, HSP60, and small heat shock proteins (sHSPs) (Wang et al., 2004). Although almost all HSPs are related to heat tolerance, sHSPs, especially sHSPs in chloroplasts and mitochondria, are presumed to be more important to heat resistance in plants because they protect the heat-sensitive photosynthetic system II protein complex from damage, ensure normal electron transport and ATP synthesis, and allow plants to grow normally under heat stress (Banzet et al., 1998; Downs and Heckathorn, 1998; Hamilton and Heckathorn, 2001; Chauhan et al., 2012; Zhong et al., 2013).

sHSPs usually contain a non-conserved N-terminal sequence (NTS), a conserved β-sandwich α-crystallin domain (ACD) of approximately 80–100 amino acid residues, and a shorter, non-conserved C-terminal sequence (CTS). The variable NTS and CTS participate in the combination of sHSP dimers into oligomers. In addition, they contain different signal peptides that determine the subcellular localization of sHSPs (Waters et al., 1996; Waters, 2013; Haslbeck and Vierling, 2015). Bioinformatics analysis indicates that plants have many more types and quantities of sHSPs than animals due to gene duplication, thus enabling sessile plants to adapt to various adverse environmental factors (Haslbeck and Vierling, 2015). sHSPs are generally not detected in plant tissues at optimal temperatures, but when plants are subjected to heat stress, the amount of sHSPs increases rapidly, even by several hundreds of times, and can make up 1% of all cellular proteins (Lindquist and Craig, 1988; Morimoto, 1993; Swindell et al., 2007). The accumulation of sHSPs is proportional to the strength and duration of the heat stress. When the heat stress is removed, the sHSPs are still highly stable, with a half-life of 30–50 h (Chen et al., 1990; Derocher et al., 1991; Sedaghatmehr et al., 2016). Therefore, sHSPs are likely to be closely related to the recovery and survival of plants after heat stress (Waters et al., 1996).

In addition to heat stress, sHSP production is also induced by other abiotic and biotic stresses, such as drought, salinity, cold and pathogens. In addition, they also participate in various developmental processes, such as embryogenesis, seed germination, sugar metabolism, photosynthesis, chloroplast development and fruit development (Sun et al., 2001, 2002). The over-expression (OE) of sHSPs in plants with transgenic technology can not only enhance the heat tolerance of transgenic plants but also improve the abilities of transgenic plants to tolerate other stresses, such as salinity, drought, heavy metals and disease. Most importantly, the OE of sHSPs in transgenic plants does not cause any adverse or defective phenotypes (Kong et al., 2014; Zhang et al., 2014; Fragkostefanakis et al., 2015; Hu et al., 2015; Khaskheli et al., 2015; Sun et al., 2016; Kuang et al., 2017). For example, the OE of a sHSP gene (*MasHSP24.4*) from wild banana into tomato significantly enhanced tomato tolerance to high temperatures and drought (Mahesh et al., 2013). Additionally, the OE of *OsHSP18.0* in a susceptible rice variety drastically enhanced its resistance not only to *Xanthomonas oryzae* pv. *oryzae* (Xoo) but also to heat and salt stress, whereas silencing *OsHSP18.0* in a resistant variety significantly increased its susceptibility and sensitivity to heat and salt. Therefore, the OE of *sHSP* genes plays a positive role in both biotic and abiotic defense responses (Kuang et al., 2017).

Although major progress has been made in past years, especially in clarifying the structure, evolution and function of sHSPs, the complicated heat response network and specific developmental molecular mechanisms of sHSPs are still largely unknown. To effectively screen and study the functions of heat-responsive proteins (especially sHSPs) in wheat (*Triticum aestivum L.*), isobaric tandem mass tag (TMT)-labeled quantitative proteomic analysis was performed in our previous work (Lu et al., 2017; Wang X. et al., 2018). Here, we reported that a sHSP in wheat, TaHSP23.9, was highly upregulated at both the RNA and protein levels in wheat leaves and filling grains under heat stress. Amino acid sequence analysis showed that heat-responsive TaHSP23.9 is an HSP20/ACD domain-containing chaperone that belongs to the sHSP (HSP20) family and that it is highly homologous with the *Oryza sativa* subsp. *japonica* (rice) protein OsHSP23.2. Moreover, a transient expression assay in *Nicotiana benthamiana* epidermal cells demonstrated that TaHSP23.9 is located on the endoplasmic reticulum. The OE of *TaHSP23.9* in transgenic *Arabidopsis* enhanced tolerance to heat and salinity stress, suggesting that *TaHSP23.9* positively regulates plant responses to heat and salt stress. Meanwhile, we found that the expression level of *TaHSP23.9* is much higher in heat-tolerant wheat varieties than in heat-sensitive wheat varieties. Thus, we suggest that *TaHSP23.9* could be used as a candidate gene for developing new heat-tolerant wheat varieties through genetic engineering and could be developed as a molecular marker for screening heat-tolerant wheat varieties.

## Materials and Methods

### Plant Materials and Growth Conditions

All wheat (*Triticum aestivum*) varieties used in this study are listed in Supplementary Table S1. The full-length CDS of *TaHSP23.9* was amplified from cDNA of the wheat variety “CS.” The *Arabidopsis thaliana* ecotype Columbia-0 (*Col-0*) was used as the wild type (WT). The growth conditions of wheat and *Arabidopsis* plants were the same as in our previous work (Wang et al., 2011; Wang J. et al., 2018; Lu et al., 2017). Briefly, wheat plants were grown in a greenhouse at day/night temperatures of 24/17°C and a 14/10 h day/night cycle. The *Arabidopsis* plants were grown in a growth chamber at a temperature of 22°C and a 16/8 h day/night cycle.

### Heat Stress Treatments

The heat stress treatments for wheat plants were the same as in our previous work (Lu et al., 2017; Wang J. et al., 2018). Briefly, wheat plants were first grown in the greenhouse under day/night temperatures of 24/17°C and a 14/10 h day/night cycle. When they were 10 days old or 14 days after anthesis, heat stress treatments were applied to the plants with uniform growth. The wheat plants were transferred into a growth chamber and exposed to day/night temperatures of 38/34°C and a 14/10 h day/night cycle for 0, 0.5, 1, 3, 6, 12, 24, 48, or 72 h, and their leaves (10-day-old seedlings) and filling grains (14 days after anthesis) were harvested and kept at −80°C before RNA extraction. For *Arabidopsis*, the plants were grown in a growth chamber. When they were 2 weeks old, heat stress treatments were applied to plants with uniform growth. *Arabidopsis* plants were exposed to 45°C for 0, 1, 2, 4, or 6 h, and their leaves were collected for the determination of the contents of MDA, total soluble protein and proline.

### Salt Stress Treatments

Surface-sterilized seeds of *Arabidopsis* were planted in a line on vertical 1/2 MS plates containing 1% sucrose and 0.8% agar. After 4 days, 10 healthy seedlings of each of the WT and over-expressors with approximately equal root lengths were transferred to another new vertical 1/2 MS plates containing 1% sucrose, 0.8% agar and different concentrations of Sodium chloride (0, 50, 75, 100, 125, and 150 mM). The positions of the root tips were marked by a marker pen. After 2 weeks, images were photographed, and the root elongation was measured by NIH-Image software^1^. Leaves were collected for determination of the content of MDA, total soluble protein and proline. For wheat plants, seedlings were cultured hydroponically in 1/2 Hoagland’s liquid medium until they were reached the three-leaf stage, then 200 mM NaCl was added, and the leaves of seedlings were harvested at 0, 10, 15, 30 min, 1, 3, 6, 12, 24, 48, and 72 h and kept in −80°C before RNA extraction.

### Measurement of MDA, Total Soluble Protein and Proline Content

The malondialdehyde (MDA) content was measured by using the thiobarbituric acid (TBA) reaction according to Buege and Aust (1978) with slight modifications (Buege and Aust, 1978). Briefly, approximately 0.2 g fresh leaves were fully ground with a small amount of quartz sand and 2 ml cold phosphate buffer in a cold mortar and transferred to a 15 ml tube. Cold phosphate buffer was added to a final volume of 5 ml, and this mixture was mixed fully with 5 ml 0.5% TBA reagent (0.5%, m/v TBA dissolved in 10%, m/v TCA). The mixture was kept in a boiling water bath for 10 min, quickly cooled on ice, and then centrifuged at 4°C, 3000 *g*, for 15 min. Two milliliters of supernatant was collected, and the absorbance was determined at 450 (A_450_), 532 (A_532_), and 600 nm (A_600_). The MDA content was calculated as follows:

MDAcontent(μM/g)=[6.452*(A-532A)600-0.559*A]450*

$$\left(\mathrm{Vt}/{\mathrm{Vs}}^{*}\,\mathrm{FW}\right)$$

where Vt is the total volume of the extract, Vs is the volume of extract taken during the measurement and FW is the fresh weight.

For the total soluble protein measurement, fresh leaves (approximately 0.2 g) were fully ground with 2 ml distilled water in a cold mortar and transferred to a 10 ml tube. Distilled water was added to a final volume of 5 ml, and the mixture was centrifuged at 5000 *g* for 10 min. Then, 0.1 ml supernatant was added to a 10 ml tube, to which 0.9 ml distilled water and 5 ml Coomassie brilliant blue G-250 were added. The mixture was mixed fully and incubated for 2 min, and the absorbance was determined at 595 nm. The protein concentration (C) was determined from a standard curve by using bovine serum albumin as a standard according to Bradford (1976) and the protein content was calculated as follows:

$$\mathrm{Protein}\,\mathrm{content}\,\left(\mathrm{mg}/g\right)=\left(C^{*}\,\mathrm{Vt}\right)/\left({\mathrm{FW}}^{*}\,{\mathrm{Vs}}^{*}\,1000\right)$$

where Vt is the total volume of the extract, Vs is the volume of extract taken during the measurement and FW is the fresh weight.

The proline content was measured as described by Bates et al. (1973). Briefly, approximately 0.5 g fresh leaves were added to a 10 ml tube with 5 ml aqueous sulfosalicylic acid and kept in a boiling water bath for 15 min. After cooling, 2 ml of filtrate was reacted with 2 ml acid-ninhydrin and 2 ml of glacial acetic acid in a 10 ml tube and kept in a boiling water bath for 30 min. After cooling, the absorbance was read at 520 nm. The proline concentration (C) was determined from a standard curve, and the proline content was calculated as follows:

$$\mathrm{Proline}\,\mathrm{content}\,\left(\mu \,g/g\right)=\left(C^{*}\,\mathrm{Vt}\right)/{\left({\mathrm{FW}}^{*}\,\mathrm{Vs}\right)}^{*}\,100\%$$

where Vt is the total volume of the extract, Vs is the volume of extract taken during the measurement and FW is the fresh weight.

### RT-PCR and Quantitative Real-Time PCR (qRT-PCR)

Total RNA extraction, *RT-PCR* and *qRT-PCR* were performed the same as in our previous work (Wang et al., 2011; Wang J. et al., 2018). *Actin1* mRNA (for wheat) and *Tubulin5* mRNA (for Arabidopsis) were used as an internal control and the relative expression levels of each genes were calculated based on the comparative threshold cycle (Ct). Each experiment had three biological replicates and at least three technical replicates. All primers used in RT-PCR and qRT-PCR are listed in Supplementary Table S2.

### Vector Construction and Transformation

The full-length CDS of *TaHSP23.9* was amplified by specific primers that listed in Supplementary Table S1. The PrimeSTAR HS DNA Polymerase (Takara, Dalian, China) was used for amplification, and the expected PCR fragment was cloned into pDONR/Zeo entry vector by BP recombination clonase (Invitrogen, Carlsbad, CA, United States). After the sequence was 100% matched the sequence published on the website^2^, the *TaHSP23.9* was cloned into pBIB-BASTA-35S-GWR destination vector by LR recombination clonase (Invitrogen, Carlsbad, CA, United States). The recombinant plasmid pBIB-BASTA-35S-TaHSP23.9 was introduced into the *Agrobacterium tumefaciens* strain GV3101. Transformation of *Arabidopsis thaliana* was performed by the floral dip method (Clough and Bent, 1998). The homozygous lines with single insertion were obtained from transformants that produced 100% BASTA-resistant progenies in the T_3_ generation, and three independent homozygous 35S-*TaHSP23.9* lines were selected for further experiments.

### Subcellular Localization

The coding region of TaHSP23.9 without a termination codon was cloned into the pCAM35-GFP vector, yielding a CaM35S:TaHSP23.9-GFP fusion construct. For transient co-expression, the fusion construct and ER marker mCherry-HDEL (Nelson et al., 2007) were transformed into *Nicotiana benthamiana* leaf cells according to the protocol described previously (Sparkes et al., 2006). Fluorescence signals were observed under the confocal laser scanning microscope (Leica TCS-SP4), and the parameters were as follows: GFP, excitation at 488 nm (Argon laser), scanning at 498–548 nm; mCherry-HDEL, excitation at 543 nm (Helium-Neon laser), scanning at 543–631 nm.

### Phylogenetic Analysis

The full length of *TaHSP23.9* was used as a query to search the UniProt database^2^. All the sequences of related sHSPs were downloaded and aligned using ClustalX 2.0 software (Thompson et al., 2002). The phylogenetic trees were constructed by neighbor-joining method (1000 bootstrap replicates) and drawn by MEGA 5.0 software (Tamura et al., 2011).

### Accession Numbers

All accession numbers are listed in Supplementary Table S3, and all the genes can be found by searching their corresponding accession numbers on the website https://www.uniprot.org/(UniProt).

### Statistical Analyses

All data were processed by Microsoft Excel 2010 (Microsoft Corporation^3^) and the significant analysis of differences between control and treatments were performed by IBM SPSS Statistics 23 (International Business Machines Corporation)^4^. All experiments were performed and analyzed separately at least with three biological replicates.

### TaHSP23.9 Was Upregulated by Heat Stress and Highly Expressed in Filling Grains

**FIGURE 1:**
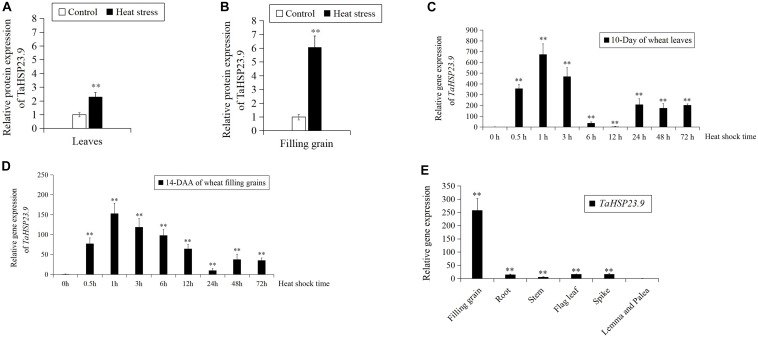
The expression pattern of TaHSP23.9 and *TaHSP23.9.* **(A)** Relative protein expression of TaHSP23.9 in the flag leaves from wheat plants (15-day after anthesis) grown under heat stress. **(B)** Relative protein expression of TaHSP23.9 in the filling grains from wheat plants (15-day after anthesis) grown under heat stress. **(C)** Relative gene expression of *TaHSP23.9* in the leaves of wheat seedlings (10-day) at different time points under heat stress (the expression level of *TaHSP23.9* under normal condition was used as the control). **(D)** Relative gene expression of *TaHSP23.9* in the filling grains from wheat plants (14-day after anthesis) at different time points under heat stress (the expression level of *TaHSP23.9* under normal condition was used as the control). **(E)** Relative gene expression of *TaHSP23.9* in different tissues from wheat plants (14-day after anthesis) under normal condition (the expression level of *TaHSP23.9* in the lemma and palea was used as the control). *TaActin1* was used as internal control for qRT-PCR. The relative expression levels of each genes were calculated based on the comparative threshold cycle (Ct). Each experiment had three biological replicates and at least three technical replicates. Error bars indicate the SD. Double asterisks above bars indicate significant differences between control and heat treatment, or significant differences among the lemma and palea with other tissues at *P* < 0.01 level (*t-test*).

In our previous work, we conducted TMT-labeled quantitative proteomic analysis in leaves and filling grains to identify heat responsive proteins (HRPs) that are involved in the wheat heat response network (Lu et al., 2017; Wang X. et al., 2018). In both experiments, the sHSP protein “W5AT01” (protein accession number, updated to “A0A3B6B0K8” on https://www.uniprot.org/, designated TaHSP23.9 based on its molecular weight in this study) was upregulated 2.3-fold in leaves (Figure 1A) and 6.1-fold in filling grains (Figure 1B) under heat stress. Then, we conducted qRT-PCR to further verify the expression pattern of *TaHSP23.9* under heat stress by using RNA samples derived from leaves and filling grains of wheat that were exposed to heat stress at 38/34°C and 14/10 h light/dark for 0 to 72 h. The expression level of *TaHSP23.9* was significantly upregulated by heat and reached a maximum at 1 h of heat stress (Figures 1C,D). These results indicate that *TaHSP23.9* is a heat*-*responsive gene and is upregulated by heat stress at both the RNA and protein levels, suggesting its important role in the HSR. In addition, we also checked the tissue-specific expression pattern of *TaHSP23.9* in wheat under normal conditions, and we found that *TaHSP23.9* was expressed in the filling grains, roots, stems, flag leaves, spikes, lemma and palea. Moreover, the expression level in the filling grains was much higher than that in the other tissues, suggesting its important role in seed development (Figure 1E).

### Cloning and Characterization of *TaHSP23.9*

**FIGURE 2:**
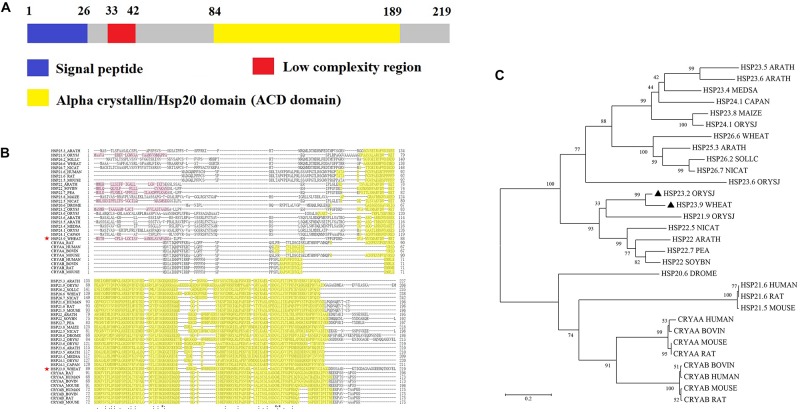
Amino acid sequence alignment and phylogenetic tree analysis of TaHSP23.9. **(A)** Schematic diagram amino acid sequence of TaHSP23.9. **(B)** Amino acid sequence alignment of TaHSP23.9 with other representative sHSPs which molecular weight about 20 to 27 Da from other species. Yellow shaded amino acids denote conserved ACD domain. The most conserved residues of ACD are indicated by asterisks, more conserved residues of ACD are indicated by dots. TaHSP23.9 is marked with a red star. **(C)** Phylogenetic tree analysis of TaHSP23.9 with other representative sHSPs which molecular weight about 20–27 Da from other species. The sequences used for phylogenetic analysis include the sHSPs from *Arabidopsis thaliana* (mouse-ear cress), *Medicago sativa* (Alfalfa), *Capsicum annuum* (Capsicum pepper), *Zea mays* (maize), *Oryza sativa subsp. japonica* (Rice), *Triticum aestivum* (Wheat), *Solanum lycopersicum* (Tomato), *Nicotiana attenuata* (Coyote tobacco), *Pisum sativum* (Garden pea), *Glycine max* (Soybean), *Drosophila melanogaster* (fruit fly), *Homo sapiens* (human), *Rattus norvegicus* (rat), *Mus musculus* (mouse), and *Bos taurus* (bovine). TaHSP23.9 from wheat and OsHSP23.2 from rice are marked with a black triangle. The phylogenetic tree was constructed by neighbor-joining method (1000 bootstrap replicates) and drawn by MEGA 5.0 software.

**FIGURE 3:**
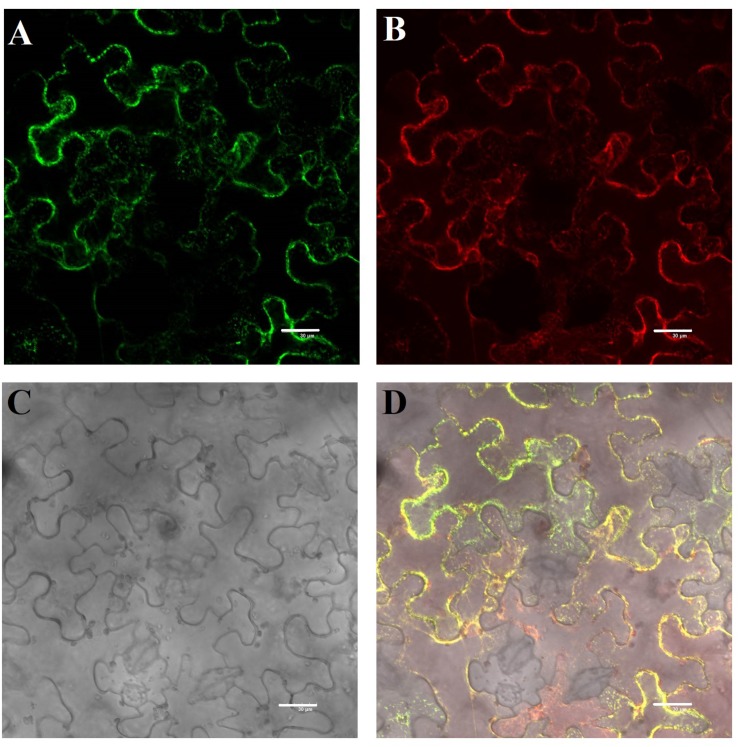
Subcellular localization of the TaHSP23.9 protein in *Nicotiana benthamiana* epidermal cells. **(A)** GFP fluorescence of 35S:TaHSP23.9–GFP. **(B)** ER marker mCherry-HDEL. **(C)** Bright-field image of the *Nicotiana benthamiana* epidermal cells. **(D)** Merged image of **(A–C)**. Scale bars = 30 μm.

We cloned the full-length CDS of *TaHSP23.9* (UniProtKB accession number: “A0A3B6B0K8”) with specific primers (Supplementary Table S2) from the cDNA of “CS” (Chinese Spring, a wheat variety). The full-length CDS of *TaHSP23.9* is 660 bp and encodes a protein of 219 amino acids. The mass of TaHSP23.9 is approximately 23,940 (Da). Alignment of the amino acid sequences of TaHSP23.9 with other representative sHSPs with molecular weights of approximately 20–27 Da from other species showed that TaHSP23.9 contains one typical structure of the alpha-crystallin domain (ACD) at amino acid residues 84–189 and a low-complexity region at amino acid residues 33–42. In addition, a signal peptide consisting of 26 amino acids was also found at the N-terminus of TaHSP23.9, and TaHSP23.9 was predicted to be localized on the endoplasmic reticulum by ProtComp v.9.0 database analysis^5^ (Figures 2A,B). Phylogenetic tree analysis indicated that TaHSP23.9 was closely related to *Oryza sativa* subsp. *japonica* (rice) HSP23.2_ORYSJ (OsHSP23.2), which is also an endoplasmic reticulum sHSP, with amino acid sequence similarities of 70.1% (Figure 2C). To confirm the subcellular localization of TaHSP23.9, we constructed a CaM35S:TaHSP23.9-GFP fusion vector and conducted a transient expression assay in *Nicotiana benthamiana* epidermal cells. As shown in Figure 3, the green signal of TaHSP23.9-GFP overlapped with the red signal of HDEL-RFP (the marker for the endoplasmic reticulum), indicated in orange. Taken together, these results demonstrate that TaHSP23.9 is a member of the plant sHSP gene family and is located on the endoplasmic reticulum.

### Over-Expression of *TaHSP23.9* in Arabidopsis Enhanced Tolerance to Heat and Salt Stress

**FIGURE 4:**
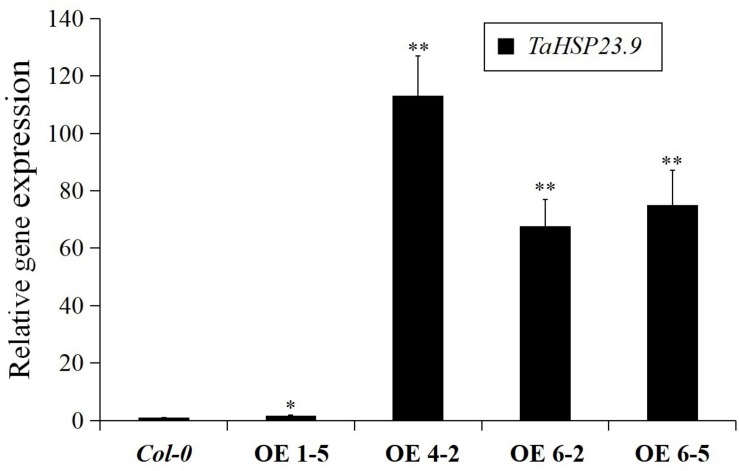
Relative gene expression of *TaHSP23.9* in the over-expression transgenic lines. Total RNA extracted from 2-week-old seedlings grown on 1/2 MS were subjected to RT–PCR analysis. *AtTubulin5* was used as internal control for qRT-PCR. The expression level of *TaHSP23.9* in *Col-0* was used as the control. The relative expression levels of each genes were calculated based on the comparative threshold cycle (Ct). Each experiment had three biological replicates and at least three technical replicates. Error bars indicate the SD. Single and double asterisks above bars indicate significant differences between *Col-0* (WT) and *TaHSP23.9*-OE lines (1-5, 4-2, 6-2, 6-5) at *P* < 0.05 level and *P* < 0.01 level (*t-test*) respectively.

**FIGURE 5:**
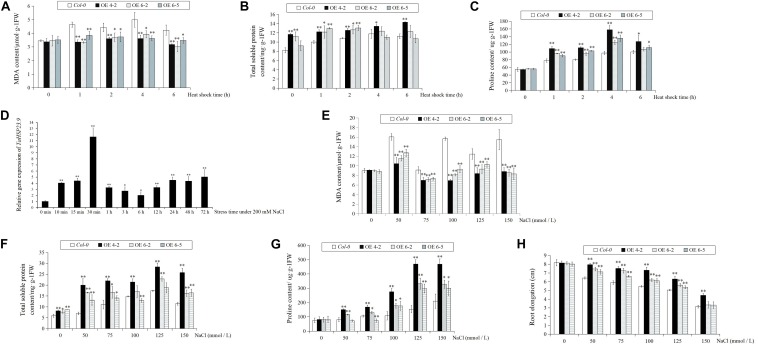
The relative expression level of *TaHSP23.9* under salt stress, and the MDA, total soluble protein, and proline contents and root elongation of *Col-0* (WT) and *TaHSP23.9* over-expression transgenic lines under heat and salt tress. **(A–C)** The contents of MDA, total soluble protein and proline in *Col-0* and *TaHSP23.9*-OE lines under heat stress. **(D)** Relative gene expression of *TaHSP23.9* in the leaves from wheat plants (three-leaf stage) at different time points under salt stress (200 mM NaCl treatment, the expression level of *TaHSP23.9* under normal condition was used as control). **(E–H)** The contents of MDA, total soluble protein and proline and the root elongation of *Col-0* and *TaHSP23.9*-OE lines under salt stress. All data were obtained from at least ten plants for each genotype. Error bars indicate the SD. Single and double asterisks above the bars indicate significant differences between the control and salt stress treatments or between the *Col-0* (WT) and *TaHSP23.9*-OE lines at *P* < 0.05 and *P* < 0.01 (*t*-test), respectively.

The *TaHSP23.9* OE transgenic lines were verified by qRT-PCR (Figure 4). Based on the expression level of *TaHSP23.9*, we selected *TaHSP23.9*-OE lines 4-2, 6-2, and 6-5 for subsequent analyses. Compared with wild-type (*Col-0*), the MDA content of the *TaHSP23.9*-OE lines was significantly lower than that of WT (Figure 5A), but the total soluble protein and proline contents of the *TaHSP23.9*-OE lines were significantly higher than those of WT under heat stress (Figures 5B,C). We also induced salt stress and found that *TaHSP23.9* is a salt*-*responsive gene upregulated by salt stress (Figure 5D). In addition, the conditions of the *TaHSP23.9*-OE lines were much better than that of WT under NaCl treatment, the MDA content of *TaHSP23.9*-OE lines was significantly lower than that of WT (Figure 5E), and the total soluble protein and proline contents of *TaHSP23.9*-OE lines were significantly higher than those of WT (Figures 5F,G). Additionally, the root elongation in the *TaHSP23.9*-OE lines was significantly longer than that of the WT under the NaCl treatment (Figure 5H and Supplementary Figure S1). Taken together, these results indicate that *TaHSP23.9* is also a salt*-*responsive gene and that OE of *TaHSP23.9* in transgenic *Arabidopsis* can enhance resistance to heat and salt stress.

### The Expression Level of *TaHSP23.9* in Heat-Tolerant Varieties Is Much Higher Than That in Heat-Sensitive Varieties

**FIGURE 6:**
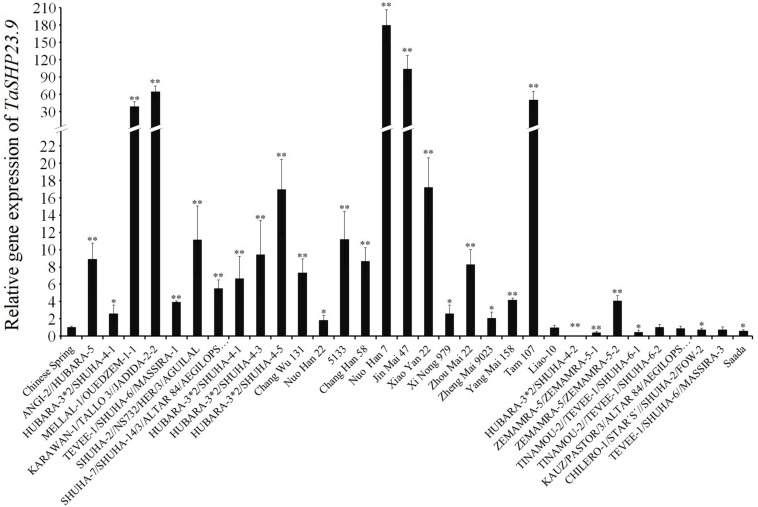
Relative gene expression of *TaHSP23.9* in the heat-tolerant wheat varieties and in the heat-sensitive varieties. Total RNA extracted from wheat leaves (three-leaf stage) were subjected to RT–PCR analysis. *TaActin1* was used as internal control for qRT-PCR. The expression of *TaHSP23.9* in the leaves of “CS” was used as the control. The relative expression levels of each genes were calculated based on the comparative threshold cycle (Ct). Each experiment had three biological replicates and at least three technical replicates. Error bars indicate the SD. Single and Double asterisks above bars indicate significant differences among “CS” and other wheat varieties at *P* < 0.05 level and *P* < 0.01 level (*t*-test) respectively.

The expression level of *TaHSP23.9* was consistent with the differences in heat tolerance among the wheat varieties, as the expression level of *TaHSP23.9* was significantly higher in the heat-tolerant wheat varieties (Liu et al., 2018; Wang X. et al., 2018) “MELLAL-1/OUEDZEM-1-1,” “KARAWAN-1/TALLO 3//JADIDA-2-2,” “Nuo Han 7,” “Jin Mai 47,” and “TAM107” than that in the heat-sensitive wheat varieties “CS,” “HUBARA-3^∗^2/SHUHA-4-2,” “ZEMAMRA-5/ZEMAMRA-5-1,” and “Saada” under normal conditions (Figure 6). Therefore, we suggest that *TaHSP23.9* could be developed as a molecular marker for screening heat-tolerant wheat varieties; it could also be used as a candidate gene for improving the heat tolerance of wheat by transgenic technology.

### Over-Expression of *TaHSP23.9* in Transgenic Plants Did Not Cause Any Adverse Phenotypes or Significantly Change the Expression of Abiotic Stress-Responsive Genes Under Normal Conditions

**FIGURE 7:**
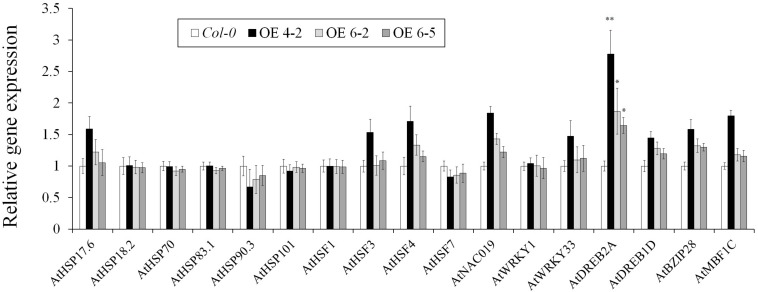
Relative expression levels of heat responsive genes and other stress-related genes in *Col-0* (WT) and *TaHSP23.9* over-expression transgenic lines. Total RNA extracted from 2-week-old seedlings grown on 1/2 MS were subjected to RT–PCR analysis. *AtTubulin5* was used as the internal control for qRT-PCR. The expression level of these genes in *Col-0* was used as the control. The relative expression level of each gene was calculated based on the comparative threshold cycle (Ct). Each experiment had three biological replicates and at least three technical replicates. Error bars indicate the SD. Single and double asterisks above the bars indicate significant differences between the *Col-0* (WT) and *TaHSP23.9*-OE lines (4-2, 6-5) at *P* < 0.05 and *P* < 0.01 (*t*-test), respectively.

No visible differences or adverse phenotypes were observed between the wild-type (*Col-0*) and *TaHSP23.9*-OE lines under normal growth conditions (Supplementary Figure S2). The comparison of the relative expression levels of some heat- and other abiotic stress-related genes (including *AtHSP17.6*, *AtHSP18.2*, *AtHSP70*, *AtHSP83.1*, *AtHSP90.3*, *AtHSP101*, *AtHSF1*, *AtHSF3*, *AtHSF4*, *AtHSF7*, *AtNAC019*, *AtWRKY1*, *AtWRKY33*, *AtDREB2A*, *AtDREB1D*, *AtBZIP28*, and *AtMBF1C*) (Lim et al., 2006; Meiri and Breiman, 2009; Fragkostefanakis et al., 2015; Ohama et al., 2017) in the WT and *TaHSP23.9*-OE lines showed that the relative expression levels of almost all these genes were not significantly different between the wild-type and *TaHSP23.9*-OE lines, except that of one gene, *AtDREB2A* (Figure 7). This indicates that the OE of *TaHSP23.9* does not cause dramatic transcriptome changes or adverse phenotypes that could lead to reduced yield and/or poor quality in transgenic plants.

## Discussion

sHSPs are a class of proteins that are synthesized by prokaryotic and eukaryotic organisms in response to heat stress or other environmental stresses (Sun et al., 2002). Although sHSPs do not modulate heat stress directly in plants, they play an important role in thermotolerance as molecular chaperones to maintain proteostasis. sHSPs are generally active as large oligomers consisting of multiple subunits and are believed to be ATP-independent chaperones that can protect other proteins against heat-induced denaturation and aggregation. Meanwhile, they are important in refolding in combination with other HSPs (Wang et al., 2004; Waters, 2013; Haslbeck and Vierling, 2015). Although many studies have confirmed the positive role of sHSPs against environmental stresses in different plant species, the molecular mechanisms for these multiple functions are still unknown.

In this study, we cloned the heat-responsive gene *TaHSP23.9* from wheat based on the TMT-labeled quantitative proteomic analysis in our previous work (Lu et al., 2017; Wang X. et al., 2018) and examined its function in transgenic *Arabidopsis*. Bioinformatics analysis of this protein showed that TaHSP23.9 has one ACD domain. This indicates that it is evolutionarily related to alpha-crystallin, which is widely distributed in different tissues where it exerts its biological function as a chaperone. A low-complexity region at amino acid residues 33–42 may be involved in the flexible binding that determines both its binding properties and its biological roles (Coletta et al., 2010); in addition, a signal peptide at the N-terminus determines its subcellular localization to the endoplasmic reticulum (Figures 2, 3). The endoplasmic reticulum, which is widely present in eukaryotic cells, is an important site for protein synthesis, folding, assembly, and transport and plays a critical role in integrating signals generated by both biotic and abiotic stress in plants (Park and Park, 2019). Therefore, we presume that the endoplasmic reticulum sHSPs as well as chloroplast and mitochondrial sHSPs are more important and more closely related to heat tolerance than other sHSPs for maintaining proteostasis and protecting proteins against heat-induced denaturation in plants. The expression levels of sHSPs were different under normal and stress conditions. Usually, the expression levels of most sHSPs are moderate, and some sHSPs are not expressed under normal conditions. However, under stress conditions, the expression level of sHSPs is increased dramatically (Morimoto, 1993; Sun et al., 2002; Swindell et al., 2007). Compared with other tissues we tested, the expression level of *TaHSP23.9* was much higher in the filling grains under normal conditions (Figure 1E), and *TaHSP23.9* was quickly and significantly over-expressed by HS (Figures 1C,D). This suggests its important role in seed development and HSR, which maintained the proteostasis of grains during the filling stage and the milk stage (the late growth stage of wheat) when subjected to high temperatures.

TaHSP23.9 was identified as a heat-responsive protein in our previous work (Lu et al., 2017; Wang X. et al., 2018). Therefore, we first verified its gene expression pattern and function in transgenic *Arabidopsis* under heat stress. The qRT-PCR results were consistent with the TMT-labeled quantitative proteomic analysis, indicating that *TaHSP23.9* can be upregulated by heat stress at both the mRNA and protein levels (Figures 1A–D). This suggests that *TaHSP23.9* plays an important role in HSR. A large number of studies have shown that the contents of the harmful substance MDA and the protective substances soluble protein and proline are greatly accumulated when plants are subjected to various stress conditions; thus, the contents of these three substances are often used as physiological indexes to determine plant resistance to various stresses (Hasegawa et al., 2000; Gill and Tuteja, 2010; Szabados and Savoure, 2010; Krasensky and Jonak, 2012). When over-expressing *TaHSP23.9* in transgenic *Arabidopsis*, we found that the transgenic plants were more resistant to heat stress than wild-type plants, with lower MDA contents and higher total soluble protein and proline contents (Figures 5A–C). Therefore, we concluded that *TaHSP23.9* plays a positive role in the HSR and acts as a chaperone to repair damage caused by heat stress. In addition, many studies have confirmed that sHSPs can be induced not only by heat stress but also by various stress environments, and OE of sHSPs in transgenic plants can enhance plant tolerance to multiple stresses (Zou et al., 2012; Mu et al., 2013; Zhang et al., 2014, 2018; Kuang et al., 2017). Therefore, we verified the phenotype of *TaHSP23.9-OE* lines and the expression pattern of *TaHSP23.9* under NaCl treatment to examine whether *TaHSP23.9* participates in salt stress responses. The results indicated that *TaHSP23.9* is also a salt-responsive gene that can be significantly upregulated by salt (Figure 5D), and the OE of *TaHSP23.9* in transgenic *Arabidopsis* can also enhance resistance to salt stress (Figures 5E–H and Supplementary Figure S1). Hence, we concluded that *TaHSP23.9* may act as a chaperone and play a positive role in the response to both heat and salt stress.

The thermotolerance of crops is a complex quantitative trait that is controlled by multiple genes and is vulnerable to environmental factors (Maphosa et al., 2014). Meanwhile, there is a lack of direct and stable evaluation indicators or evaluation index systems, and the evaluation period usually occurs at the terminal stage of wheat development, for example, by evaluating wheat spikelet fertility or the seed setting rate and plumpness at high temperatures. Given these constraints, it is time-consuming and ineffective to screen heat-tolerant wheat varieties by traditional methods. Therefore, it is urgent and necessary to identify and develop molecular markers for screening wheat genotypes with different thermotolerances. Chen et al. (2014) identified five sHSPs (OsHSP16.9A, OsHSP17.4, OsHSP17.9A, OsHSP23.2, and OsHSP26.7) that were upregulated by heat stress, and the expression level of these five sHSPs was closely correlated with the thermotolerance of different rice varieties, suggesting that these five genes can be used as molecular markers for screening heat-tolerant rice cultivars (Chen et al., 2014). Interestingly, TaHSP23.9 was closely related to OsHSP23.2 (Figure 2C), one of the five sHSPs, with amino acid sequence similarities of 70.1% identified through amino acid alignment and phylogenetic tree analysis. This suggests its potential application as a molecular marker for screening heat-tolerant wheat genotypes. Indeed, we found that the expression level of *TaHSP23.9* was significantly higher in the young leaves of heat-tolerant wheat varieties than in the young leaves of heat-sensitive varieties under normal conditions (Figure 6). This result will assist us in promoting the efficiency of the screening and identification of heat-tolerant wheat varieties at the early stage of wheat development. Thus, *TaHSP23.9* could be developed as a molecular marker for screening heat-tolerant wheat varieties.

Finally, we observed the phenotypes of the *TaHSP23.9-OE* lines carefully and found no visible differences or adverse phenotypes in transgenic plants or wild-type plants under normal conditions (Supplementary Figure S2), and resistance to heat and salt was enhanced under the heat stress and NaCl treatments. Therefore, we checked the expression levels of some heat- and other abiotic stress-related genes (Lim et al., 2006; Meiri and Breiman, 2009; Fragkostefanakis et al., 2015; Ohama et al., 2017) to determine whether their transcription levels were changed significantly between wild-type and *TaHSP23.9-OE* lines under normal conditions. The results indicated that almost all the genes we examined were not significantly changed except for *AtDREB2A* (Figure 7). *AtDREB2A* is one of the DREB TFs that extensively participates in various stress responses and interacts with a *cis*-acting dehydration-responsive element (DRE) sequence to activate the expression of downstream genes that are involved in salt, drought, low temperature and HSRs (Lata and Prasad, 2011). However, until now, the mechanism of activation of DREB2-type genes has not been well studied. The OE of *AtDREB2A* (Liu et al., 1998) and *OsDREB2A* (Dubouzet et al., 2003) did not induce target stress-induced genes. Therefore, we suggest that the OE of *TaHSP23.9* in transgenic *Arabidopsis* plants does not cause dramatic transcriptome changes or adverse phenotypes that could lead to reduced yield and/or poor quality in transgenic plants, and *TaHSP23.9* could be used as a candidate gene for improving the thermotolerance in wheat with transgenic technology.

sHSPs are a class of proteins with low molecular weights that are easy to clone and genetically transform in crops. Moreover, the OE of sHSPs in plants through transgenic technology not only enhances their resistance to biotic and abiotic stresses but also does not cause any adverse phenotypes that could lead to reduced yield and/or poor quality in transgenic crops. The completion of the sequencing of the wheat genome will accelerate wheat molecular biology research and assist us in understanding the HSR network and elucidating the distinct mechanism of each wheat sHSP gene in response to HS. With this understanding, improving the thermotolerance of wheat using transgenic technology as well as screening and identifying heat-tolerant wheat varieties by molecular markers will become much more convenient.

## Data Availability Statement

All datasets generated for this study are included in the article/Supplementary Material.

## Author Contributions

JW, XG, YF, and ZW designed the study, performed the data analyses, and wrote the manuscript. JZW cloned the gene. JD and XT performed physiological and qRT-PCR experiments JW performed the subcellular localization of the TaHSP23.9. JP and SX revised the manuscript. All authors have read and approved the final manuscript.

## Conflict of Interest

The authors declare that the research was conducted in the absence of any commercial or financial relationships that could be construed as a potential conflict of interest.

## Footnotes

1 https://imagej.nih.gov/ij/index.html

2 https://www.uniprot.org/

3 www.microsoft.com

4 www.ibm.com/products/spss-statistics

5 http://linux1.softberry.com/berry.phtml?topic=protcomppl&group=programs&subgroup=proloc

## Abbreviations

ACD: alpha-crystallin domain
FW: fresh weight
HRPs: heat responsive proteins
HS: heat stress
HSF: heat shock transcription factor
HSP: heat shock protein
HSR: heat stress response
OE: over-expression
sHSP: small heat shock protein
WT: wild type.
