# Supplementary material for: Over-Expression of the Heat-Responsive Wheat Gene TaHSP23.9 in Transgenic Arabidopsis Conferred Tolerance to Heat and Salt Stress
Source: Front Plant Sci. 2020 Mar 6;11:243. doi: 10.3389/fpls.2020.00243 (PMC7069362; doi:10.3389/fpls.2020.00243)
Supplement: Supplementary file 1 [file Data_Sheet_1.PDF]

Over-expression of the heat-responsive wheat gene *TaHSP23.9* in transgenic *Arabidopsis* conferred tolerance to heat and salt stress

Jun Wang[1#], Xin Gao[1#], Jun Dong[1], Xinyu Tian[1], Junzhe Wang[1], Jairo A Palta[2,3], Shengbao Xu[1], Yan Fang[4*], and Zhonghua Wang[1*]

1. Supplementary Figures and Tables

1.1 Supplementary Figures

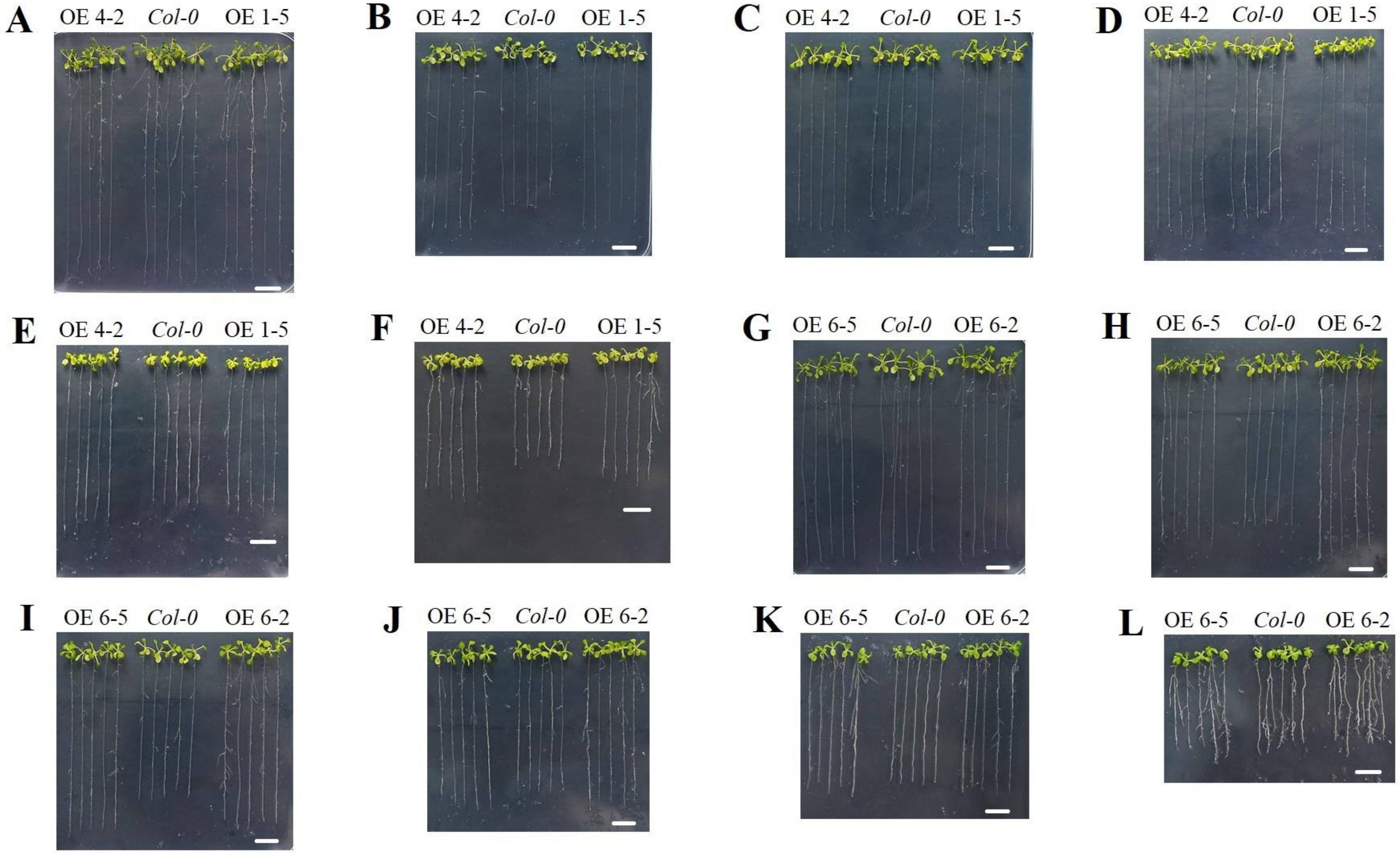
A
OE 4-2 Col-0 OE 1-5
B
OE 4-2 Col-0 OE 1-5
C
OE 4-2 Col-0 OE 1-5
D
OE 4-2 Col-0 OE 1-5
E
OE 4-2 Col-0 OE 1-5
F
OE 4-2 Col-0 OE 1-5
G
OE 6-5 Col-0 OE 6-2
H
OE 6-5 Col-0 OE 6-2
I
OE 6-5 Col-0 OE 6-2
J
OE 6-5 Col-0 OE 6-2
K
OE 6-5 Col-0 OE 6-2
L
OE 6-5 Col-0 OE 6-2

Supplementary Figure 1. Phenotype of *Col-0* and *TaHSP23.9*-OE lines under NaCl treatment. (A) to (F) the concentration of NaCl was 0 mM, 50 mM, 75 mM, 100 mM, 125 mM and 150 mM respectively. (G) to (L) the concentration of NaCl was 0 mM, 50 mM, 75 mM, 100 mM, 125 mM and 150 mM respectively. Scale bars = 1 cm

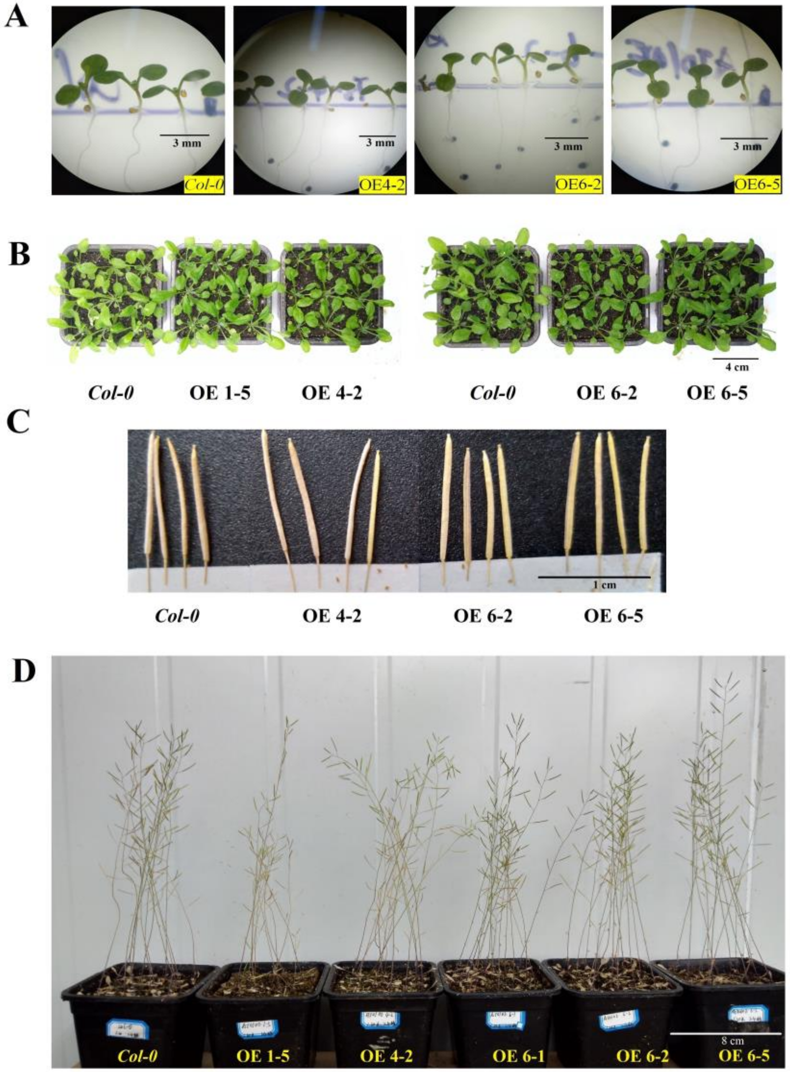

A
3 mm
Col-0
OE4-2
OE6-2
OE6-5
B
4 cm
Col-0
OE 1-5
OE 4-2
Col-0
OE 6-2
OE 6-5
C
1 cm
Col-0
OE 4-2
OE 6-2
OE 6-5
D
8 cm
Col-0
OE 1-5
OE 4-2
OE 6-1
OE 6-2
OE 6-5

Supplementary Figure 2. Phenotype of *Col-0* and *TaHSP23.9*-OE lines under normal condition. (A) Seedlings of 4 DAG (Days After Germination), sale bar = 3 mm. (B) Seedlings of 21 DAG, sale bar = 4 cm. (C) Matured siliques, sale bar = 1 cm. (D) Matured plants, sale bar = 8 cm.

1.2 Supplementary Tables

Supplementary Table 1. Wheat (*Triticum aestivum*) varieties used in this study

**Name**
Chinese Spring (CS)
Tam 107
HUBARA-3*2/SHUHA-4-5
ANGI-2//HUBARA-5
HUBARA-3*2/SHUHA-4-3
HUBARA-3*2/SHUHA-4-1
MELLAL-1/OUEDZEM-1-1
SHUHA-7/SHUHA-14/3/ALTAR 84/AEGILOPS SQUARROSA(TAUS)//OPATA-3
SHUHA-2//NS732/HER/3/AGUILAL
HUBARA-3*2/SHUHA-4-1
KARAWAN-1/TALLO 3//JADIDA-2-2
TEVEE-1/SHUHA-6//MASSIRA-1
TEVEE-1/SHUHA-6//MASSIRA-3
TINAMOU-2//TEVEE-1/SHUHA-6-1
CHILERO-1/STAR´S´//SHUHA-2/FOW-2
TINAMOU-2//TEVEE-1/SHUHA-6-2
KAUZ/PASTOR/3/ALTAR 84/AEGILOPS SQUARROSA-1
ZEMAMRA-5/ZEMAMRA-5-1

HUBARA-3*2/SHUHA-4-2
Saada
Liao-10
ZEMAMRA-5/ZEMAMRA-5-2
Chang Wu 131
Nuo Han 22
5133
Chang Han 58
Nuo Han 7
Jin Mai 47
Xiao Yan 22
Xi Nong 979
Zhou Mai 22
Zheng Mai 9023
Yang Mai 158

Supplementary Table 2. Primers used in this study

**Name**	**Primer (5′-3′)**
**Primers for cloning and constructing *TaHSP23.9***	
TaHSP23.9-F	AAAAAGCAGGCTTCATGTCGACTCGATGCTTCTTGGC
Ta HSP23.9-R	AGAAAGCTGGGTCTCAGAGCTCGACTTTCTTGGTC
AttB1 (adaptor)	GGGGACAAGTTTGTACAAAAAAGCAGGCTTC
AttB2 (adaptor)	GGGGACCACTTTGTACAAGAAAGCTGGGTC
**Primers for qRT-PCR**	
TaHSP23.9-F	GAGACCTCCGACTCCCAC
TaHSP23.9-R	CGCCCTTCCGTTCTTCCA

TaACTIN1-F	GTATCGCTGACCGTATGA
TaACTIN1-R	GACACTGTACTTCCTCTCA
AtTUBULIN5-F	CACAGGAGAAGGAATGGA
AtTUBULIN5-R	TCATCAGCAGTAGCATCT
AtHSP17.6-F	GGTGAGTGGCAAAAGACAGA
AtHSP17.6-R	AAACTTCCCCATCCTCCTCT
AtHSP18.2-F	TTACCGGAGAATGCAAAGATG
AtHSP18.2-R	CGGAGATATCGATGGACTTGA
AtHSP70-F	AGGAGCTCGAGTCTCTTTGC
AtHSP70-R	AGGTGTGTCGTCATCCATTC
AtHSP83.1-F	GCTGCTAGGATTCACAGGATG
AtHSP83.1-R	TCCTCCATCTTGCTCTCTTCA
AtHSP90.3-F	GAGTATGCTATTGGTCAG
AtHSP90.3-R	CTTCTTCTTCTCGTCTTC
AtHSP101-F	TAACGGGCCAAAGAGAAGTG
AtHSP101-R	CACACGTTGGAGGTCAAGACT
AtHSF1-F	AATGCCAGAGACTTCCCAGAT
AtHSF1-R	AATGGGCTTGGAGAGATGAAT
AtHSF3-F	CAAGAACCAAACGAGTGGACCCGTAA
AtHSF3-R	TCCTTTTGCAACCTCCCCTGAAAATC
AtHSF4-F	GGACCGGGATGAAAAGAATTA
AtHSF4-R	CACGCTGGTTTGAACAGTCTT
AtHSF7-F	TGGAGGAGAATAACTCCGGTAA
AtHSF7-R	ATGCAATGGGGATTCAGTAACA
AtNAC019-F	AGTATCGTCTCATTGAACCT
AtNAC019-R	CACTTGATTGCTTCTTGTATATTC

AtWRKY1-F	ATGATGTTGTTGCCACTA
AtWRKY1-R	CTTACAGTCAGTAGATTCCA
AtWRKY33-F	AACTACACTACTTCTTCTCA
AtWRKY33-R	GTTGCTGTTGTTGTTATTG
AtDREB2A-F	CTCAACTACGGAATACCT
AtDREB2A-R	CAGATCCAAGTAACTCAAG
AtDREB1D-F	ATGATGTTGCTGCTTTAG
AtDREB1D-R	AGTAGTCTCAGGAATACG
AtBZIP28-F	GGTATTACGATGAACATAAG
AtBZIP28-R	TAATCTACTCCTCCACAA
AtMBF1C-F	GCGAGATTGGAGAAGAAG
AtMBF1C-R	CATATTCCTGAACTACCTGAG

Supplementary Table 3. Accession numbers in this study

**Gene name**	**Accession number in UniProt database**
HSP25.3_ARATH	P31170
HSP21.9_ORYSJ	Q53M11
HSP26.2_SOLLC	Q95661
HSP26.6_WHEAT	Q00445
HSP26.7_NICAT	A0A1J6ITS1
HSP21.6_HUMAN	Q9UJY1
HSP21.6_RAT	Q9EPX0
HSP21.5_MOUSE	Q9JK92
HSP22_ARATH	Q38806
HSP22_SOYBN	P30236
HSP22.7_PEA	P19244

HSP23.8_MAIZE	O64960
HSP22.5_NICAT	A0A1J6KW68
HSP20.6_DROME	P02516
HSP23.2_ORYSJ	Q7XUW5
HSP23.6_ORYSJ	B7EZJ7
HSP23.6_ARATH	Q96331
HSP23.5_ARATH	Q9FGM9
HSP23.4_MEDSA	A0A1C6ZYA7
HSP24.1_ORYSJ	Q6Z7V2
HSP24.1_CAPAN	D9IAX1
HSP23.9_WHEAT	A0A3B6B0K8
CRYAA_RAT	P24623
CRYAA_HUMAN	P02489
CRYAA_BOVIN	P02470
CRYAA_MOUSE	P24622
CRYAB_HUMAN	P02511
CRYAB_BOVIN	P02510
CRYAB_RAT	P23928
CRYAB_MOUSE	P23927
AtHSP17.6	Y14070
AtHSP18.2	X17295
AtHSP70	AJ002551
AtHSP83.1	M62984
AtHSP90.3	AT5G56010
AtHSP101	AF218796
AtHSF1	X76167

AtHSF3	Y14068
AtHSF4	Y14069
AtHSF7	AJ251868;
AtNAC019	AT1G52890
AtWRKY1	AT2G04880
AtWRKY33	AT2G38470
AtDREB2A	AT5G05410
AtDREB1D	AT5G51990
AtBZIP28	AT3G10800
AtMBF1C	AT3G24500
